# Detecting Depression Signs on Social Media: A Systematic Literature Review

**DOI:** 10.3390/healthcare10020291

**Published:** 2022-02-01

**Authors:** Rafael Salas-Zárate, Giner Alor-Hernández, María del Pilar Salas-Zárate, Mario Andrés Paredes-Valverde, Maritza Bustos-López, José Luis Sánchez-Cervantes

**Affiliations:** 1 Tecnológico Nacional de México/I. T. Orizaba, Av. Oriente 9 No. 852, Col. Emiliano Zapata, Orizaba 94320, Veracruz, Mexico; dci.rsalas@ito-depi.edu.mx; 2 Tecnológico Nacional de México/I.T.S. Teziutlán, Fracción I y II S/N, Aire Libre, Teziutlán 73960, Puebla, Mexico; maria.sz@teziutlan.tecnm.mx (M.d.P.S.-Z.); mario.pv@teziutlan.tecnm.mx (M.A.P.-V.); 3 Centro de Investigación en Inteligencia Artificial/Universidad Veracruzana, Sebastián Camacho 5, Zona Centro, Centro, Xalapa-Enríquez 91000, Veracruz, Mexico; maritbustos@gmail.com; 4 CONACYT-Tecnológico Nacional de México/I. T. Orizaba, Av. Oriente 9 No. 852, Col. Emiliano Zapata, Orizaba 94320, Veracruz, Mexico; jlsanchez@conacyt.mx

**Keywords:** depression, social media, sentiment analysis

## Abstract

Among mental health diseases, depression is one of the most severe, as it often leads to suicide; due to this, it is important to identify and summarize existing evidence concerning depression sign detection research on social media using the data provided by users. This review examines aspects of primary studies exploring depression detection from social media submissions (from 2016 to mid-2021). The search for primary studies was conducted in five digital libraries: ACM Digital Library, IEEE Xplore Digital Library, SpringerLink, Science Direct, and PubMed, as well as on the search engine Google Scholar to broaden the results. Extracting and synthesizing the data from each paper was the main activity of this work. Thirty-four primary studies were analyzed and evaluated. Twitter was the most studied social media for depression sign detection. Word embedding was the most prominent linguistic feature extraction method. Support vector machine (SVM) was the most used machine-learning algorithm. Similarly, the most popular computing tool was from Python libraries. Finally, cross-validation (CV) was the most common statistical analysis method used to evaluate the results obtained. Using social media along with computing tools and classification methods contributes to current efforts in public healthcare to detect signs of depression from sources close to patients.

## 1. Introduction

Mental disorders are a worldwide health problem affecting a large number of people and causing numerous deaths every year. According to a World Health Organization (WHO) report, the most common major disorders in 2017 included anxiety (284 million sufferers), depression (264 million), bipolar disorder (46 million), schizophrenia (20 million), and eating disorders (16 million) [1].

According to the American Psychiatric Association (APA), depression is a serious and common medical condition that negatively affects how people feel and act and the way they think. Fortunately, major depression is also treatable. Depression is an important factor in suicide among both adolescents and the elderly, but those with a late onset of depression are at higher risk [2]. In fact, nearly 800,000 people die due to suicide every year, and suicide alone is the second leading cause of death among 15–29 year-old people (WHO). Depression can lead to physical and emotional problems and can affect a person’s ability to work [3]. Furthermore, the stress factors of the COVID-19 crisis indicate that a great number of people in the world may be in the course of developing depression as a result of the new and unusual lifestyle caused by the pandemic. It is also common for the effects of a viral disease to affect people’s moods, causing them to go into depressive states; moreover, the COVID-19 crisis has increased the chances of depression, which in turn will make recovery from the pandemic harder across a spectrum of needs [4]. According to Szmuda [5], during the current situation, telemedicine and social media allow patients to receive healthcare while still practicing social distancing, the principal anti-pandemic defense. Moreover, bots can be adjusted quickly based on the latest research findings and WHO recommendations on COVID-19. With triage being exclusively handled by bots, nurses and clinicians can devote more of their time to patient care. We can say that the focus of this research is valuable in the application of tools to detect the onset of depressive problems in people so that they can be used in healthcare institutions, as well as in the support of individuals, making those who suffer from mental problems more participatory in relation to their mental health. When the period of social isolation finishes, people suffering from depression will have a harder time returning to their common social activities and exercise, and when the virus infection abates, people with depression are more likely to suffer from immunological problems, making them more prone to other conditions [6].

During this time, it is crucial for psychiatrists to become familiar with screening and triage procedures and work closely with public health specialists and physicians to reduce the problems that their patients face [7].

The study of social media, particularly in the public health domain, is a rapidly growing research area. For instance, social media are commonly used to monitor outbreaks of infectious diseases [8,9,10,11] and understand trends in prescription medication usage [12]. Furthermore, several authors [13,14,15,16] claim that the value of social media in understanding mental health is of the utmost importance, since they provide access to the public accounts, behaviors, activities, thoughts, and feelings of users that may be indicative of their emotional wellbeing.

Since social media information is of great value for identifying people at risk of depression or with other mental disorders, many models and systems have been developed to detect the signs and symptoms of mental illnesses from social media data. For instance, Renara et al. [17] found that sentiment analysis on social media could help monitor the mood of a person, which is particularly important since people with depression symptoms experience similar feelings and have similar behavior, which are often expressed through what they post on their social media platforms. To perform sentiment analysis, the n-gram model, i.e., a set of n consecutive words, is commonly used. In fact, several authors [18,19,20,21] use the n-gram model for the specific case of n equals one (*n* = 1), which is also called unigram. According to De Choudhury and Gamon [13], the following unigrams are associated with depression signs or symptoms: retraction, psychosis, harsh, delusions, ADHD, imbalance, sleeplessness, suicidal, vertigo, retching, attacks, sleep, seizures, addictive, weaned, swings, dysfunction, appetite, fuzzy, irritability, episodes, headache, tiredness, edging, anxiety, burden, heaviness, and somnolent. On the other hand, investigations from these authors [22,23,24,25] have demonstrated the results obtained in this topic. From this perspective, it seems relevant for the scientific community to perform a systematic literature review to identify and become familiar with the social media sites and features of datasets, methods for linguistic feature extraction, machine-learning algorithms, computing tools, and statistical analysis methods currently employed to determine depression on social media.

The scope of this research is to identify and summarize the existing evidence concerning depression sign detection on social media via computing tools, methods for linguistic feature extraction, statistical analysis techniques, and machine-learning algorithms. The research follows the methodology proposed by Brereton et al. [26] to review relevant literature from the last five years (from 2016 to mid-2021), which were retrieved from major academic digital libraries. Then, we synthetize the results from our primary sources using strategies for reducing bias and random errors. Our findings highlight the social media sites, computing tools, methods for linguistic feature extraction, statistical analysis techniques, and machine-learning algorithms most used in depression sign detection research. We also analyze and discuss literature reviews similar to ours to emphasize the progress being made in terms of depression sign detection via innovative techniques. The review is focused on the research into depression sign detection and seeks to elucidate the different methods used for detecting depression on social media using sentiment analysis.

### An Overview of Machine-Learning Techniques, Dataset Features, and Social Media

Sentiment analysis (SA) is a technique for analyzing consumer opinions and producing data that can depict these opinions as a whole [27]. SA is also known as opinion mining, a text analysis technique that analyzes the opinions of human emotions toward entities and the features that exist in these entities [28]. In the context of SA, a feature is an item that people talk about in relation to services, products, policies, events, organizations, or individuals. The combination of features and corresponding sentiment words can help produce accurate, meaningful, and high-quality sentiment analysis results [27].

Machine-learning (ML) techniques are applied in sentiment classification to organize text into positive, negative, or neutral categories. Training datasets and testing datasets are used in ML techniques. The training datasets are applied to learn the documents, while the testing datasets are used to validate the execution of ML techniques [29]. As Maetschke et al. [30] explain, machine-learning algorithms comprise supervised, unsupervised, and semisupervised methods. Unsupervised methods are applied on expression data but have a lower prediction capability than supervised methods. Supervised methods need data on known associates for training, and these are often scarce. Semisupervised methods can be trained with fewer interaction data but are generally less accurate predictors than supervised methods.

Social media allows researchers to obtain behavioral data relevant to a person’s way of thinking, emotional state, communication, activities, and means of relating. The texts that are published on social networks allow the detection of feelings of uselessness, guilt, powerlessness, and self-aversion that determine the signs of depression. According to De Choudhury and Gamon [13], changes in social relationships, activity, and language can be applied to build statistical models that allow the detection and prediction of depression in a more precise way, including ways that can complement traditional diagnostic approaches.

The rest of this paper is organized as follows: Section 2 discusses the goal and justification of the research, while Section 3 explains the methods, which include our research questions, search strategy, selection process of primary studies, and data extraction process. The results of the review are included in Section 4, whereas in Section 5 we introduce a discussion of the results. At the end, in Section 6 we define the conclusions and suggestions for future work.

## 2. Research Goal and Need for Literature Review

This literature review seeks to identify and summarize existing evidence concerning depression sign detection research on social media using methods of linguistic feature extraction, machine-learning algorithms, computing tools, and statistical analysis methods. Currently, there are works that address a theme similar to that of this work. Table 1 lists research works similar to ours, for example, Guntuku et al. [31] focus on studies aimed at predicting mental illness using social media. First, they consider the methods used to predict depression, and then they consider four approaches that have been used in the literature: prediction based on survey responses, prediction based on self-declared mental health status, prediction based on forum membership, and prediction based on annotated posts. Wang et al. [32] examined relevant investigations with the Beck Depression Inventory-II for measuring depression in medical settings to provide guidelines for practicing clinicians. The Beck Depression Inventory-II showed high reliability and good correlation with the measures of depression and anxiety. Its threshold for detecting depression varied according to the type of patient, suggesting the need for adjusted cutoff points. The somatic and cognitive–affective dimension described the latent structure of the instrument. Gottlieb et al. [33] showed that contextual interventions for the prevention and treatment of depressive symptoms and psychological distress can be effective, though very limited data exist in this field. Policy implications include a greater emphasis on improving conditions to decrease the incidence of depression and other mental disorders.

Although the aforementioned works share some similarities with our research, none of them review sentiment-analysis-based initiatives. Moreover, only one of the works reviewed social media for predicting mental illnesses, but it did not specifically focus on depression sign detection. From this perspective, we conclude that the principal differences between our literature review and similar works are as follows: (1) we analyze the most recent relevant works; (2) we identify the social media sites most commonly studied and the features of the datasets retrieved; and we determine (3) the linguistic feature extraction methods, (4) machine-learning algorithms, (5) computing tools, and (6) mathematical analysis methods most commonly applied in depression sign detection from social media.

## 3. Methods

**Figure 1:**
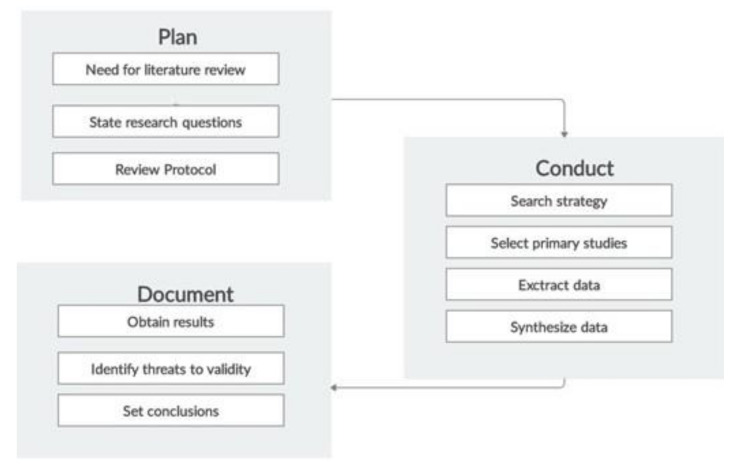
Literature review process.

This literature review examines quantitative and qualitative aspects of primary studies exploring depression detection from social media submissions via novel approaches and methods. We followed the three-stage methodology depicted in Figure 1, which was proposed by Brereton et al. [26] as a straightforward method for conducting systematic literature reviews. The planning stage of the methodology comprises three steps: (a) determine need for literature review, (b) state research questions, and (c) review the protocol. Next, the conducting stage of the methodology comprises four steps: (a) determine search strategy, (b) select primary studies, (c) extract data, and (d) synthesize data. In the end, the documenting stage involves three steps: (a) obtain results, (b) identify threats to validity, and (c) establish conclusions.

### 3.1. Research Questions and Motivations

Five research questions were formulated that oriented the research and helped meet the objectives of the review. These questions are listed in Table 2.

### 3.2. Search Strategy

The search for primary studies was conducted in five digital libraries: ACM Digital Library, IEEE Xplore Digital Library, SpringerLink, Science Direct, and PubMed, as well as on the search engine Google Scholar to broaden our results. We selected the libraries based on their prestige and popularity in the scientific community, since they all provide access to a large proportion of digital literature, especially peer-reviewed articles, on a wide range of topics, including those related to our research. In a second step, we conducted a search based on keywords. To do this, we performed two tasks: we first identified a set of words or phrases in relation to our search topic (i.e., keywords); then, we identified related concepts. As for the search period, our review was intended to be not only accurate, but also up to date. To this end, the search covered the last six years—from 2016 to mid-2021. Finally, regarding the keyword search, Table 3 lists the set of keywords and related concepts used.

**Figure 2:**
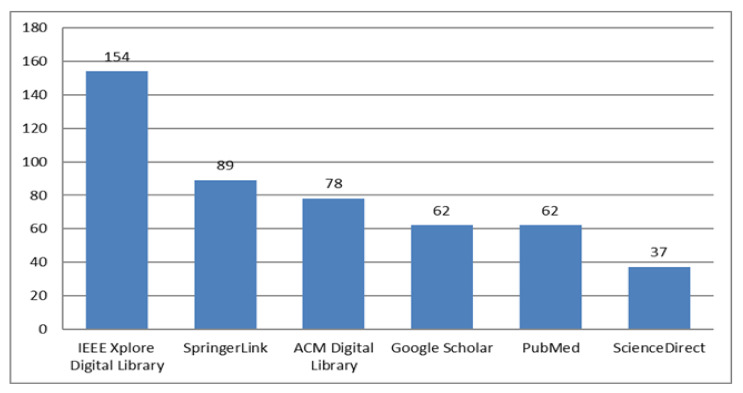
Research papers by digital libraries.

The search strings were formed by combining the keywords listed in Table 3 using connectors “AND” and “OR” as follows: ((Depression) OR (Mental Health) OR (Mental illness) OR (Mental disorder) AND (Social media OR Social networks OR Social web OR Microblogs OR Twitter OR Facebook OR Reddit OR Instagram OR Weibo OR NHANES)) Year: 2016–2021. As Figure 2 shows graphically, we found 482 relevant search results: 154 from IEEE Xplore Digital Library, 89 from SpringerLink, 78 from ACM Digital Library, 62 from Google Scholar, 62 from PubMed, and 37 from ScienceDirect.

According to Figure 2, the majority of the literature regarding depression detection on social media is produced by IEEE, followed by SpringerLink and ACM. Conversely, Google Scholar and PubMed provide access to fewer research articles on the subject matter. Finally, we found lowest number of publications relevant to our search on Science Direct.

### 3.3. Selection of Primary Studies

We selected only studies including at least one of the keywords such as *Depression*, *Social Media,* and related concepts (see Table 3).

We identified 420 records through database searching; furthermore, we identified 62 additional records through other sources such as Google Scholar. After the duplicates were removed, we obtained 287 papers that determined the records screened. Once we had read the abstracts, were excluded 95 (57 master and doctoral dissertations and 38 papers not written in English). Then, we read the full articles assessed for eligibility and excluded 158 studies conducted in domains other than detecting depression signs on social media to obtain the studies included in the synthesis (192). Finally, we obtained 34 studies that constituted the studies included in the quantitative synthesis.

**Figure 3:**
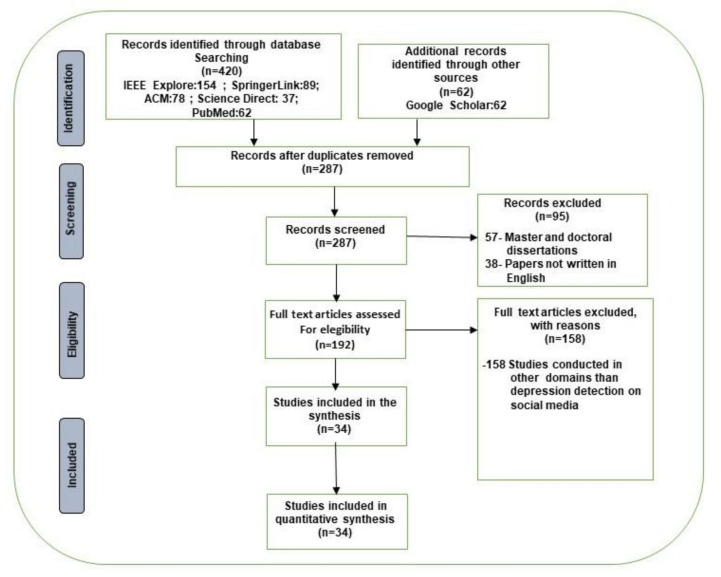
PRISMA flow diagram for the literature search.

A PRISMA diagram [34] is shown in Figure 3 that represents the flow diagram of the papers searched and chosen for our review.

**Figure 4:**
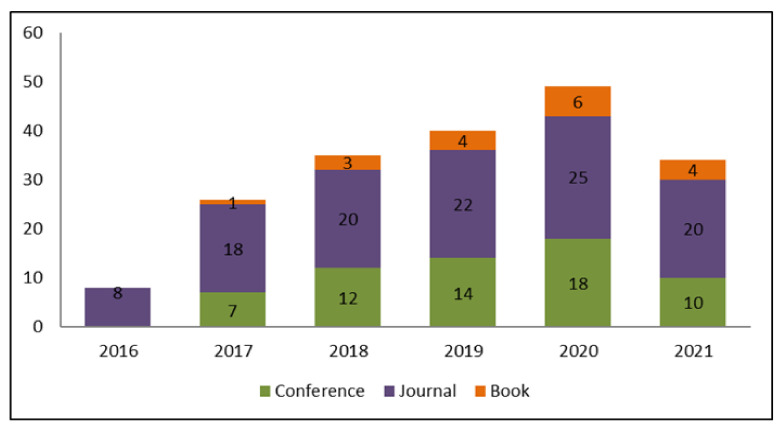
Type of publication from 2016 to mid-2021.

We retrieved and analyzed 192 full text articles assessed for eligibility but only considered 34 primary studies. As depicted in Figure 4, 59% of the retrieved publications were published in journals, 32% in conference proceedings, and 9% as book chapters. As regards the year of publication, 8 papers were issued in 2016 (journals); 26 papers were published in 2017 (7 in conference proceedings, 18 in journals, and 1 as a book chapter); 35 papers were published in 2018 (12 in conference proceedings, 20 in journals, and 3 as book chapters); 40 were issued in 2019 (14 in conference proceedings, 22 in journals, and 4 as book chapters); 49 papers were published in 2020 (18 in conference proceedings, 25 in journals, and 6 as book chapters); and finally, 34 papers were published in the first half of 2021 (10 in conference proceedings, 20 in journals, and 4 as book chapters).

**Figure 5:**
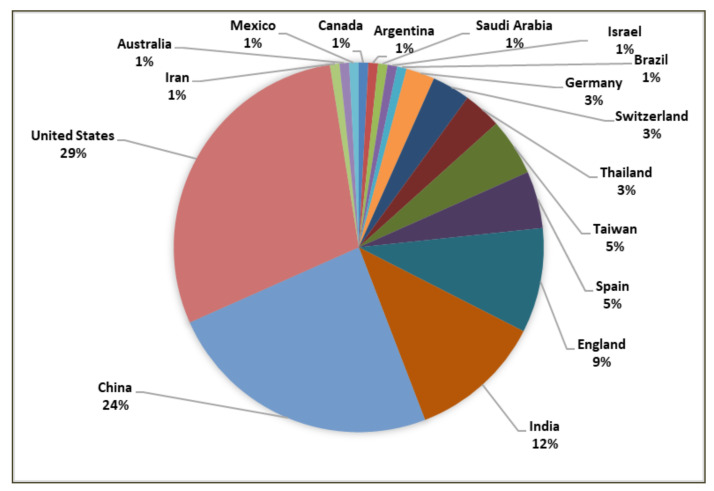
Geographical distribution.

Figure 5 graphically represents the geographical distribution of the retrieved publications. As can be seen, the majority of the research was conducted in the United States (29%), China (24%), India (12%), England (9%), Spain (5%), Taiwan (5%), Thailand (3%), Switzerland (3%), Germany (3%), Brazil (1%), Israel (1%), Saudi Arabia (1%), Argentina (1%), Canada (1%), Mexico (1%), Australia (1%), and Iran (1%).

### 3.4. Data Extraction

We retrieved two types of data from the papers: bibliographic data and content data. The former included information such as research title, author names, research goal, and research database; the latter concerned actual information on the research, namely, the studied social media sites and dataset features, along with the computing tools, linguistic feature extraction models, mathematical analysis methods, and machine-learning algorithms used for depression sign detection. The following section discusses our findings.

## 4. Results

As previously mentioned, we initially retrieved 192 relevant works but ultimately selected and reviewed 34 primary studies, which better described the researched topic. The findings of the review are discussed in the following five subsections, corresponding to our five research questions. The first subsection discusses the most common social media sites and corresponding features of datasets used for depression detection on social media. In the second subsection, we discuss linguistic feature extraction methods from sentiment analysis found in the literature. Then, in the third subsection, we discuss the machine-learning algorithms most commonly applied when trying to detect depression signs from social media data, whereas the fourth subsection identifies the most common computing tools used to process the data. Finally, the fifth subsection reviews the main statistical analysis methods used to validate the results of the classification algorithms applied.

### 4.1. RQ1: Which Are the Main Social Media Sites and Dataset Features Used in Depression Detection?

Table 4 lists the social media sites and features of datasets most commonly studied in depression detection research during the period of 2016 to mid-2021.

**Figure 6:**
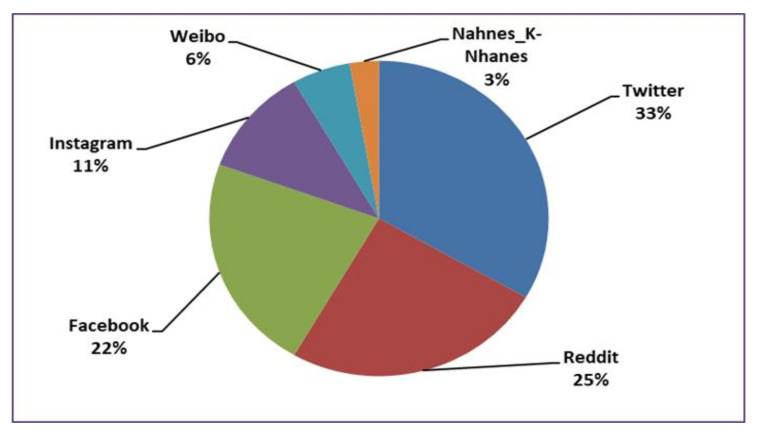
Social media sites explored in depression sign detection research.

According to Table 4 and Figure 6, Twitter, Reddit, and Facebook—in that specific order—are the social media sites most commonly studied. In the case of Twitter, the study of Leis et al. [35] was applied to texts in Spanish and was developed in two steps. In the first step, the selection of users and the compilation of tweets were performed. A total of three datasets of tweets were created, a depressive users dataset (made up of the timeline of 90 users who explicitly mentioned that they suffer from depression), a depressive tweets dataset (a manual selection of tweets from the previous users, which included expressions indicative of depression), and a control dataset (made up of the timeline of 450 randomly selected users). In the second step, the comparison and analysis of the three datasets of tweets were carried out.

In the case of Reddit, Rissola et al. [48] introduced a methodology to automatically gather post samples in English of depression and nondepression and used the dataset to train models which are able to determine whether a post conveys evidence of depression.

Katchapakirin et al. [56] employed Natural Language Processing (NLP) techniques to develop a depression detection algorithm for the Thai language on Facebook, which people use as a tool for sharing opinions, feelings, and life events. Results from 35 Facebook users indicated that Facebook behaviors could predict depression level.

Instagram is less prominently researched form of social media, since the platform emphasizes photograph and video sharing rather than text sharing, although some researchers have focused on the alternative text descriptions from Instagram posts to develop their research. We also found a few social media sites that are distinctive to a particular region. For instance, Weibo was studied in China by Li et al. [66], and K-NHANES and NHANES in Korea and the US, respectively, by Oh et al. [68]. Some of these studies were designed to be applied among speakers of other languages, such as Chinese, Thai, Korean, Arabic, and Portuguese. Overall, our findings indicate a growing use of social networking services around the globe.

### 4.2. RQ2: Which Are the Main Linguistic Feature Extraction Methods Used for Detecting Depression Signs on Social Media?

Table 5 lists our findings in response to the second research question.

Methods for linguistic feature extraction are important since researchers need to use basic elements to determine whether a person shows or does not show depression symptoms. As can be observed from Table 5, word embedding is a prominent model used to detect depression from social media data. In word embedding, each word from a text is listed as a continuous, low dimensional, and real-valued vector [58], and researchers may combine word embedding with other methods for better results. For instance, Rissola et al. [48] combined word embedding with the bag-of-words model to build a depression-post classifier using depression-positive sample posts (D+); depression-negative sample posts (D−); unigrams; word count; and the polarity scores, sadness scores, and happiness scores of words.

The n-gram model is another effective tool in depression sign research. According to Damashek [69], in the n-gram model a document can be listed as a vector whose components are the relative frequencies of its distinct constituent n-grams. In their work, Wolohan et al. [51] found that the best performing model for depression sign identification mixes word-and-character n-grams with LIWC features. As for tokenization, another model for linguistic feature extraction, Arora and Arora [39] explain that it is a process of a giving a token to a sequence of characters that we want to treat as a group; treating text as a token enables the creation of counts of tokens, which can be used as features. In the work of Aldarwish [60], the tokenize operator splits the text of a document into a sequence of tokens. For instance, the research of Tadesse et al. [50] reports the use of tokenization for data preprocessing in order to divide social media posts into individual tokens. Next, all the URLs are divided by punctuation and stop words. Then, the researchers applied stemming to decrease the words to their root form and join similar words together. As for the bag-of-words model, Nadeem [42] describe it as an approach that uses the frequency of word occurrence to determine the content of a tweet. In the bag-of-words model used by Rissola et al. [48], each post is depicted with the raw frequency of the unigrams from the textual content of the posts.

According to Arora and Arora [39], the stemming model for linguistic feature extraction refers to the process of grouping words that are close in meaning. In the study of Arora and Arora [39], the goal was to remove the suffix of a word to retrieve its base form, thus reducing redundancy. In the process of feature extraction, stemming is regularly combined with tokenization. Emotion analysis, behavior feature extraction, polarity, and POS tagging are less frequently used to detect depression from social media. As Shen et al. [36] claim, an emotion analysis determines whether the emotional state of depressed users differs from that of common users. Authors Shen et al. [37] studied emotion-related words and extracted positive and negative word counts from recent tweets using LIWC. As for the behavior feature extraction model, its usefulness is related to the fact that depression sufferers are inclined to focus on themselves and detach from others; moreover, they rarely succeed at communicating with others. Researchers Ramirez-Esparza et al. [70] performed behavior feature extraction on social media posts to identify the behavior of depression sufferers. Additionally, Wu et al. [58] applied this model with POS tagging, UKW (unknown word), word embedding, content-based features, and living-environment features.

In the polarity model, emotions can be tied to the sentiment polarity of a message defined by the text. In their research, Liu and Liu [28] consider that the negative polarity of social media posts (i.e., a value below zero) is a good indicator of unhappiness or distress, especially when the posts come from users with depression. In their work, Rissola et al. [48] combined the polarity score, word count, happiness score, and sadness score of social media posts to build a depression predictor model. Finally, POS tagging is a form of syntactic analysis with countless applications in Natural Language Processing (NLP). According to Lovins [71], it is also one of the most basic parts of the linguistic pipeline.

### 4.3. RQ3: Which Are the Main Machine-Learning Algorithms Used for Detecting Depression Signs on Social Media?

To respond to this question, Table 6 lists our review of the machine-learning algorithms used in depression sign detection research.

Machine-learning algorithms are powerful generalizers and predictors [72]. According to Baharudin et al. [73], many algorithms and techniques have been recently proposed for the classification and clustering of digital documents.

According to Batta [74], Support Vector Machines are supervised learning models with associated learning algorithms that analyze data used for classification and regression analysis. In addition to performing linear classification, SVMs can efficiently perform a nonlinear classification using what is called the kernel trick, implicitly mapping their inputs into high-dimensional feature space. Ray [75] explains that logistic regression is used to deal with classification problems. It gives a binomial outcome for the probability of whether or not an event will occur (in terms of 0 and 1), based on the values of input variables. For example, predicting whether a tumor is malignant or benign or an e-mail is classified as spam or not. Logistic regression deals with the prediction of target variables that are categorical. According to Batta [74], a neural network is a series of algorithms that endeavors to recognize underlying relationships in a set of data through a process that mimics the way the human brain operates. In this sense, neural networks refer to systems of neurons, either organic or artificial in nature. Neural networks can adapt to changing input; thus, the network generates the best possible result without needing to redesign the output criteria.

Related to our review, machine-learning algorithms increase the accuracy of predictions in multiple types of datasets. In some cases, several algorithms are used in a single research work. For example, Leiva and Freire [47] use support vector machine, logistic regression, random forest, k-nearest neighbor, linear regression, and ensemble classifiers; Rissola et al. [48] use support vector machine and logistic regression.

**Figure 7:**
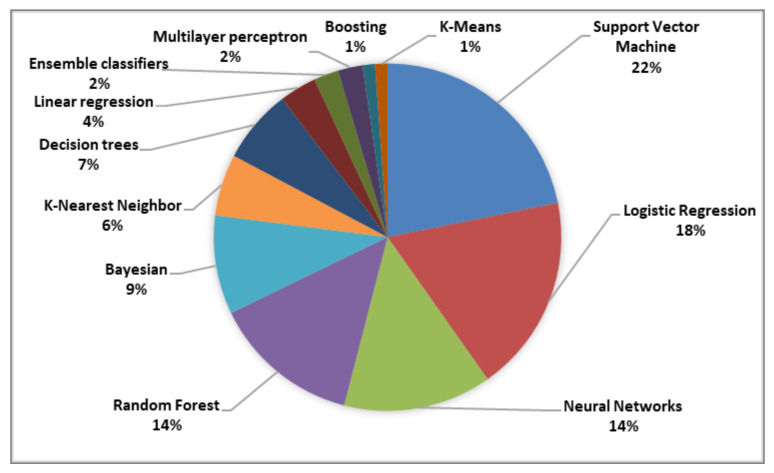
Machine-learning algorithms used for detecting depression signs on social media.

As can be observed from Figure 7, researchers generally rely on SVM, logistic regression, or neural networks to complete their diagnosis of depression from social media data. Other machine-learning algorithms less frequently employed include random forests (14%), Bayesian statistics (9%), decision trees (7%), k-nearest neighbor classifiers (6%), linear regression (4%), ensemble classifiers (2%), multilayer perceptron (2%), and boosting and k-means (1%).

### 4.4. RQ4L: Which Are the Main Computing Tools Used for Detecting Depression Signs on Social Media?

To respond to this question, Table 7 shows the main computing tools used for detecting depression signs on social media.

**Figure 8:**
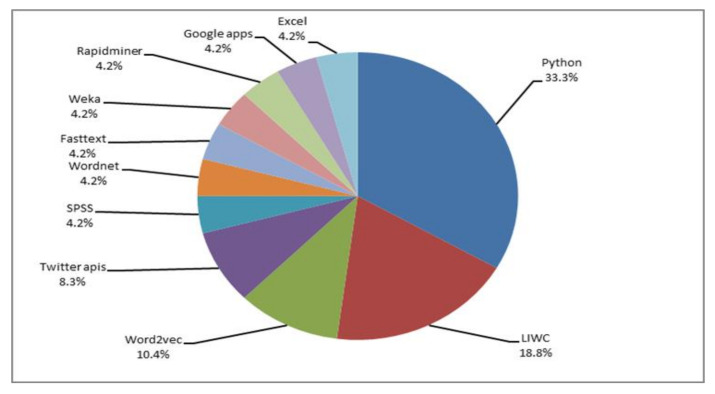
Computing tools used for detecting depression signs on social media.

Figure 8, below, introduces a graphic representation of the most common computing tools used for detecting depression signs from social media data. As can be observed, the authors use Python in first place; for example, Rissola et al. [48] use the TextBlob2 Python library to compute the polarity score of the posts in negative samples and sort them in ascending order. In the study of Leyva and Freire [47], the implementation of the learning algorithms and the vectorization were implemented with the scikit-learn library, version 0.18, for Python. In second place is LIWC (Linguistic Inquiry and Word Count). Tausczik and Pennebaker [76] explain that LIWC is a program for text analysis that counts words in psychologically meaningful categories. In their work, Shen et al. [37] extracted positive and negative word counts in recent tweets with LIWC, while Tadesse et al. [50] explored the users’ linguistic usage in the posts, employing the LIWC dictionary. Word2vec and Twitter APIs are also popular but less commonly used, followed in the list by WordNet; FastText; Weka; RapidMiner; Google Apps (in this case, it is interesting to mention that this program was used as a language translator with the Google Cloud Translation API [56]); and Microsoft Excel [60]. In the case of Microsoft Excel, the supervised dataset used in the two classifiers were created using three columns: the first being the sentiment (depressed or not depressed); the second being the depression category, which consists of one of the nine depression categories; and the third containing the manually trained posts. Finally, much less prominent tools include SPSS, Clickworker (a crowdsourcing platform), Instagram Graph API, Java, Jade, Google Cloud Translation API, and MATLAB. All these are applied along with mathematical analysis methods and machine-learning algorithms for higher accuracy in the results. Herein lies the importance of knowing which computing tools can be applied in combination with other methods.

### 4.5. RQ5: Which Are the Main Statistical Analysis Methods Used to Validate Results in Detecting Depression Signs on Social Media?

Our findings summarized in Table 8 respond to our fifth research question.

Statistical analysis is the use of mathematics to analyze data. According to our review, and as summarized in Table 8, the most common statistical analysis methods applied to validate results in depression detection research from social media include cross-validation (CV), term frequency/inverse document frequency (TF–IDF), and Cohen’s kappa statistic. On the one hand, CV is remarkably versatile; it is applicable to a wide range of problems across multiple areas. For instance, CV has been used for smoothing parameters in nonparametric smoothing and for variable selection in regression. The idea behind this method is simply splitting the data into two parts, applying the first part to determine a prediction rule, and then assessing the quality of the prediction by matching its outputs with the rest of the data; hence, the name cross-validation [77]. In the work of Ricard et al. [62], the mean and SD of the text-based scores for the most recent *k* posts were utilized as features in their model training, with *k* as a hyperparameter tuned through cross-validation. Wongkoblap et al. [57] created a predictive model and used n-fold cross-validation to report the performance of the model. The results of the evaluation are presented with accuracy, precision, recall, and the f1-score achieved by the model after training and testing with five-fold cross-validation. Oh et al. [68] ran 10-fold cross-validation for all algorithms and datasets to validate the performance of each classifier and to avoid overfitting. On the other hand, TF–IDF is a statistic used to determine the relevance of a search query to a document in a collection of documents or the occurrences of a given query in a document. It is commonly used as a basic weighting factor for text retrieval [78]. In their work, Tadesse et al. [50] used the term frequency/inverse document frequency (TF–IDF) as a numeric statistic for n-gram modelling, where the importance of a word with respect to each document in the corpora is highlighted. The main goal of its usage is to scale down the impact of empirically less-informative tokens that occur frequently to provide space for the more informative words occurring in a smaller fraction.

Finally, Cohen’s kappa statistic is a measure for assessing the degree of agreement between evaluators for the absence or presence of a trait [79]. In the work of Yazdavar et al. [43], the dataset used provided the users’ profile information, including screen name, profile description, follower/followee counts, profile image, and tweet content, which could express various depression-relevant characteristics and determine whether a user indicated any depressive behavior. They reported the inter-rater agreement as K = 0.74, based on Cohen’s kappa statistics.

Other common mathematical analysis methods include mean/standard deviation, the Mann–Whitney U test, Likert scales, and SoftMax functions, which help improve the accuracy of the results. We also found evidence of the use of variance analysis, the alternating direction method of multipliers (ADMM), Adam optimization, and Pixel-level weighted averaging.

## 5. Discussion

Depression sign detection from social media data is a growing area of interest, as the literature confirms. Data sources may vary across studies (e.g., Twitter, Facebook, Reddit, Instagram, Weibo, and NHANES). Users tend to employ social media to write about how they feel according to their interest in doing so and the facility of the use of such social media; however, in our study, we could see that much of the research into this is based on the tools that are most commonly used worldwide and that the datasets examined range from a few tweets to millions of posts. As new social media services constantly emerge, their focus continues to vary. Nowadays, a growing number of social networking services focus more on photo and video sharing rather than text sharing, thus making mental disease prediction efforts more challenging. As internet tools become more user-friendly, an increasing number of people join the social media community every day. In our study, we could see that there have been many different methods applied by researchers to extract data from tweets or posts written by users. These tools can be combined to gain better results. Machine-learning algorithms allow for the classification and clustering of data. Such tools are helpful in the process of obtaining precise results. Some authors use several of these tools in combination to ascertain which is the best for the study in question. Computer tools are necessary to process the information obtained. They perform an essential task in the sense that they help to obtain natural language information and translate or process the data to be classified. Many authors use a wide range of mathematical analysis methods; in our study, we could see that these statistical tools are useful to validate results for the detection of depression from social media. All the studies explored in this review were written in English, which is considered as the language of global scientific understanding. However, some of these studies were designed to be applied among speakers of other languages, such as Chinese, Thai, Korean, Arabic, and Portuguese.

## 6. Conclusions and Future Work

The objective of this review work was to identify all the tools necessary to detect signs of depression via social media. Using social media along with computing tools and increasingly efficient classification methods contributes to current efforts to detect signs of depression or any other mental illness from sources close to patients. This is important because, with the advance in technology, more and more people are using new media to communicate and to share experiences in the treatment of mental illnesses. Some of the studies we considered were applied in real environments and demonstrated the benefit of the research’s application in real life situations. Depression diagnosis from social media data is being widely explored around the world using a variety of networking sites, datasets, linguistic feature extraction methods, machine-learning algorithms, computing tools, and statistical analysis methods. The results obtained in most of the research works indicate that the use of new digital tools related to mental health is an incentive to continue investigating in this area. Finally, we believe that this work paves the way for further exploration of initiatives for diagnosing other mental illnesses, such as anxiety, in the sense that most of the symptoms presented in anxiety are also presented in depression. Additionally, researchers can go beyond by exploring current efforts in the monitoring and treatment of mental disorders using the Internet of Things.

## Figures and Tables

**Table 1:** Summary of related studies.

Study Reference	Approach	Year	Studies Reviewed	Years Covered
Guntuku et al. [31]	Predictive models	2017	12	2013–2017
Wang and Gorenstein [32]	Beck Depression Inventory-II	2013	70	1996–2012
Gottlieb et al. [33]	Social contexts	2011	30	1997–2008

**Table 2:** Research questions.

Research Question (RQ)	Question
RQ1	Which social media sites and features of datasets are mainly used in depression sign detection research?
RQ2	Which are the main linguistic feature extraction methods used for detecting depression signs on social media?
RQ3	Which are the main machine-learning algorithms used in depression sign detection from social media?
RQ4	Which are the main computing tools applied in detecting depression signs on social media?
RQ5	Which are the main statistical analysis methods used to validate results in detecting depression signs on social media?

**Table 3:** Keywords and related concepts of the literature review.

Area	Keywords	Related Concepts
Mental health	Depression	Mental illness
Social media	Social media	Mental disorder
		Social networks
		Social web
		Microblogs
		Twitter
		Facebook
		Reddit
		Instagram
		Weibo
		NHANES

**Table 4:** Social media and corresponding features of datasets used in depression detection research.

Social Media	Study	Features of Dataset
Twitter	Leis et al. [35]	140,946 tweets
	Kr [36]	4000+ tweets
	Shen et al. [37]	36,993 depression-candidate dataset users
	Chen et al. [38]	585 and 6596 unique and valid users with their past tweets
	Arora and Arora [39]	3754 tweets
	Biradar and Totad [40]	60,400 tweets
	Ma et al. [41]	54 million tweets
	Nadeem [42]	1,253,594 documents (tweets) as control variables
	Yazdavar et al. [43]	8770 users, including 3981 depressed users and 4789 control subjects
	Titla-Tlatelpa et al. [44]	7999 users with Twitter submissions
	Chiong et al. [45]	22191 records
	Safa et al. [46]	570 users from the control group of 16,623,164 tweets
Reddit	Leiva and Freire [47]	135 depressive users, 752 control-group users
	Rissola et al. [48]	1,076,582 submissions from 1707 unique users
	Sadeque et al. [49]	531,453 submissions from 892 unique users
	Tadesse et al. [50]	1293 depression-indicative posts, 548 standard posts
	Wolohan et al. [51]	Reddit posts from a sample of 12,106 users
	Burdisso et al. [52]	887 subjects with 531,394 submissions
	Trotzek et al. [53]	135 depressed users and a random control group of 752 users
	Titla-Tlatelpa et al. [44]	1707 users, Reddit eRisk 2018 task
	Martinez-Castaño et al. [54]	eRisk collections containing up to 1000 posts and 1000 comments
Facebook	Tai et al. [55]	3599 diaries
	Katchapakirin et al. [56]	35 Facebook users
	Wongkoblap et al. [57]	509 users in the final dataset
	Wu et al. [58]	1294 students with their data
	Yang, Mcewen, et al. [59]	22,043,394 status updates from 153,727 users
	Aldarwish and Ahmad [60]	2287 posts
	Ophir et al. [61]	190 Facebook status updates of at-risk adolescents
	Chiong et al. [45]	Facebook, Virahonda, 9178 records
Instagram	Ricard et al. [62]	data from 749 participants
	Reece and Danforth [63]	43,950 user photographs and data
	Mann et al. [64]	221 students, mean of 16.73 posts per student (60 days)
	Chun et al. [65]	520 users from Instagram through the data collection method
Weibo	Li et al. [66]	15,879 Weibo posts from 10,130 distinct Weibo users
	Lixia Yu et al. [67]	7,116,958 posts
NHANES, K-NHANES	Oh et al. [68]	dataset of 28,280 participants with 157 variables for NHANES and 4949 participants with 314 variables for K-NHANES

**Table 5:** Linguistic feature extraction methods used for detecting depression signs on social media.

Model	Study
Word embedding	Rissola et al. [48]
	Wongkoblap et al. [57]
	Wu et al. [58]
	Ma et al. [41]
	Yazdavar et al. [43]
	Trotzek et al. [53]
	Mann et al. [64]
	Titla-Tlatelpa et al. [44]
	Yueh et al. [65]
N-grams	Wolohan et al. [51]
	Rissola et al. [48]
	Sadeque et al. [49]
	Arora and Arora [39]
	Wolohan et al. [51]
	Nadeem [42]
	Titla-Tlatelpa et al. [44]
	Chiong et al. [45]
	Safa et al. [46]
Tokenization	Tadesse et al. [50]
	Arora and Arora [39]
	Biradar and Totad [40]
	Aldarwish and Ahmad [60]
	Trotzek et al. [53]
	Titla-Tlatelpa et al. [44]
	Chiong et al. [45]
	Safa et al. [46]
Bag of words	Ricard et al. [62]
	Rissola et al. [48]
	Nadeem [42]
	Mann et al. [64]
	Titla-Tlatelpa et al. [44]
	Chiong et al. [45]
	Safa et al. [46]
Stemming	Tadesse et al. [50]
	Arora and Arora [39] Aldarwish and Ahmad [60]
Emotion analysis	Leis et al. [35]
	Shen et al. [37]
	Chen et al. [38]
Part-of-Speech (POS) tagging	Wu et al. [58]
	Leis et al. [35]
	Chiong et al. [45]
Behavior features	Wu et al. [58]
	Yang, McEwen, et al. [59]
Sentiment polarity	Leis et al. [35]
	Rissola et al. [48]

**Table 6:** Machine-learning algorithms.

Machine-Learning Algorithm	Study
Support vector machine (SVM)	Leiva and Freire [47]
	Rissola et al. [48]
	Katchapakirin et al. [56]
	Sadeque et al. [49]
	Chen et al. [38]
	Tadesse et al. [50]
	Arora and Arora [39]
	Wolohan et al. [51]
	Yang, McEwen, et al. [59]
	Burdisso et al. [52]
	Li et al. [66]
	Nadeem [42]
	Yazdavar et al. [43]
	Oh et al. [68]
	Aldarwish and Ahmad [60]
	Mann et al. [64]
	Titla-Tlatelpa et al. [44]
	Chiong et al. [45]
	Safa et al. [46]
Logistic regression	Leiva and Freire [47]
	Rissola et al. [48]
	Chen et al. [38]
	Tadesse et al. [50]
	Reece and Danforth [63]
	Yang, McEwen, et al. [59]
	Burdisso et al. [52]
	Li et al. [66]
	Nadeem [42]
	Yazdavar et al. [43]
	Oh et al. [68]
	Trotzek et al. [53]
	Martinez-Cataño et al. [54]
	Chiong et al. [45]
	Safa et al. [46]
Neural networks	Kr [36]
	Sadeque et al. [49]
	Wongkoblap et al. [57]
	Wu et al. [58]
	Biradar and Totad [40]
	Yang, McEwen, et al. [59]
	Li et al. [66]
	Yazdavar et al. [43]
	Trotzek et al. [53]
	Mann et al. [64]
	Yueh et al. [65]
Random forests	Leiva and Freire [47]
	Katckapakirin et al. [56]
	Chen et al. [38]
	Tadesse et al. [50]
	Reece and Danforth [63]
	Yang, McEwen, et al. [59]
	Li et al. [66]
	Yazdavar et al. [43]
	Titla-Tlatelpa et al. [44]
	Chiong et al. [45]
	Safa et al. [46]
	Yueh et al. [65]
Bayesian statistics	Tai et al. [55]
	Chen et al. [38]
	Arora and Arora [39]
	Reece and Danforth [63]
	Yang, McEwen, et al. [59]
	Burdisso et al. [52]
	Nadeem [42]
Decision trees	Yang, McEwen, et al. [59]
	Nadeem [42]
	J Oh et al. [68]
	Titla-Tlatelpa et al. [44]
	Chiong et al. [45]
	Safa et al. [46]
K-Nearest Neighbor	Leiva and Freire [47]
	Yang, McEwen, et al. [59]
	Burdisso et al. [52]
	Oh et al. [68]
Linear regression	Leiva and Freire [47]
	Ricard et al. [62]
	Yu et al. [67]
Ensemble classifiers	Leiva and Freire [47]
	Oh et al. [68]
Multilayer Perceptron	Chiong et al. [45]
	Safa et al. [46]
Boosting	Tadeesse et al. [50]
K-Means	Ma et al. [41]

**Table 7:** Computing tools used for detecting depression signs on social media.

Computing Tool	Study
Python libraries	Kr [36]
	Leiva and Freire [47]
	Rissola et al. [48]
	Katchapakirin et al. [56]
	Tadesse et al. [50]
	Wongkoblap et al. [57]
	Biradar and Totad [40]
	Ma et al. [41]
	Burdisso et al. [52]
	Nadeem [42]
	Yazdavar et al. [43]
	Trotzek et al. [53]
	Mann et al. [64]
	Martinez-Cataño et al. [54]
	Safa et al. [46]
	Lu et al. [67]
LIWC	Shen et al. [37]
	Chen et al. [38]
	Tadesse et al. [50]
	Wolohan et al. [51]
	Yang, McEwen, et al. [59]
	Li et al. [66]
	Yazdavar et al. [43]
	Trotzek et al. [53]
	Safa et al. [46]
Word2Vec	Shen et al. [37]
	Rissola et al. [48]
	Wu et al. [58]
	Ma et al. [41]
	Yueh et al. [65]
Twitter APIs	Chen et al. [38]
	Biradar and Totad [40]
	Leis et al. [35]
	Kr [36]
WordNet	Shen et al. [37]
	Arora and Arora [39]
FastText	Rissola et al. [48]
	Trotzek et al. [53]
Weka	Katchapakirin et al. [56]
	Li et al. [66]
RapidMiner	Katchapakirin et al. [56]
	Aldarwish and Ahmad [60]
Google Apps	Katchapakirin et al. [56]
	Wu et al. [58]
Microsoft Excel	Li et al. [66]
	Aldarwish and Ahmad [60]

**Table 8:** Statistical analysis methods used to validate results in detecting depression signs on social media.

Statistical Analysis Method	Study
Cross-validation	Ricard et al. [62]
	Wongkoblap et al. [57]
	Oh et al. [68]
	Tai et al. [55]
	Sadeque et al. [49]
	Burdisso et al. [52]
	Li et al. [66]
	Nadeem [42]
	Yazdavar et al. [43]
	Mann et al. [64]
	Titla-Tlatelpa et al. [44]
	Chiong et al. [45]
Term frequency/inverse	Leiva and Freire [47]
document frequency (TF–IDF)	Tadesse et al. [50]
	Wolohan et al. [51]
	Yang, McEwen, et al. [59]
	Aldarwish and Ahmad [60]
	Martinez-Cataño et al. [54]
	Titla-Tlatelpa et al. [44]
Cohen’s kappa statistic	Rissola et al. [48]
	Li et al. [66]
	Yazdavar et al. [43]
	Yang, McEwen, et al. [59]
Mean/standard deviation	Chen et al. [38]
	Ricard et al. [62]
	Mann et al. [64]
Mann–Whitney	Ricard et al. [62]
	Ophir et al. [61]
Likert scale	Kr [36]
	Ophir et al. [61]
Softmax function	Wongkoblap et al. [57]
variance	Leis et al. [35]
Direction method of multipliers	Shen et al. [37]
Adam optimizer	Biradar and Totad [40]
Pixel-level averages	Reece and Danforth [63]

## Data Availability

Not applicable.
